# Reducing complexity: a visualisation of multimorbidity by combining disease clusters and triads

**DOI:** 10.1186/1471-2458-14-1285

**Published:** 2014-12-16

**Authors:** Ingmar Schäfer, Hanna Kaduszkiewicz, Hans-Otto Wagner, Gerhard Schön, Martin Scherer, Hendrik van den Bussche

**Affiliations:** Department of Primary Medical Care, University Medical Center Hamburg-Eppendorf, Martinistr. 52, Hamburg, 20246 Germany; Institute of General Practice, Medical Faculty, University of Kiel, Kiel, Germany; Department of Medical Biometry and Epidemiology, University Medical Center Hamburg-Eppendorf, Hamburg, Germany

**Keywords:** Multimorbidity, Multimorbidity patterns, Disease combinations, Epidemiology, Chronic diseases, Elderly people, Factor analysis, Network analysis, Observed-expected-ratios, Claims data set

## Abstract

**Background:**

Multimorbidity is highly prevalent in the elderly and relates to many adverse outcomes, such as higher mortality, increased disability and functional decline. Many studies tried to reduce the heterogeneity of multimorbidity by identifying multimorbidity clusters or disease combinations, however, the internal structure of multimorbidity clusters and the linking between disease combinations and clusters are still unknown. The aim of this study was to depict which diseases were associated with each other on person-level within the clusters and which ones were responsible for overlapping multimorbidity clusters.

**Methods:**

The study analyses insurance claims data of the *Gmünder ErsatzKasse* from 2006 with 43,632 female and 54,987 male patients who were 65 years and older. The analyses are based on multimorbidity clusters from a previous study and combinations of three diseases ("triads") identified by observed/expected ratios ≥ 2 and prevalence rates ≥ 1%. In order to visualise a "disease network", an edgelist was extracted from these triads, which was analysed by network analysis and graphically linked to multimorbidity clusters.

**Results:**

We found 57 relevant triads consisting of 31 chronic conditions with 200 disease associations ("edges") in females and 51 triads of 29 diseases with 174 edges in males. In the disease network, the cluster of cardiovascular and metabolic disorders comprised 12 of these conditions in females and 14 in males. The cluster of anxiety, depression, somatoform disorders, and pain consisted of 15 conditions in females and 12 in males.

**Conclusions:**

We were able to show which diseases were associated with each other in our data set, to which clusters the diseases were assigned, and which diseases were responsible for overlapping clusters. The disease with the highest number of associations, and the most important mediator between diseases, was chronic low back pain. In females, depression was also associated with many other diseases. We found a multitude of associations between disorders of the metabolic syndrome of which hypertension was the most central disease. The most prominent bridges were between the metabolic syndrome and musculoskeletal disorders. Guideline developers might find our approach useful as a basis for discussing which comorbidity should be addressed.

## Background

Multimorbidity is the presence of multiple chronic conditions that can include any type of disease combinations. For example, using a list of 46 chronic conditions, we found 99% of the 15,180 theoretically possible combinations of three diseases (“triads”) in a large claims data set [1]. Over the last decade, many research groups have tried to understand the complexity of multimorbidity due to its high prevalence, estimated to affect 50% to 99% of the older population [2] and its association with adverse health outcomes, such as functional status decline, lower quality of life, higher mortality risk, increased health care utilisation and, therefore, rising health care costs [3, 4]. Despite the many studies analysing the associations of diseases [5, 6], it is still unknown how to reduce the complexity of multimorbidity, e.g. in order to permit a more thorough consideration of multimorbidity in clinical practice guidelines.

In multimorbidity research, it is often assumed that health outcomes are mainly influenced by the individual diseases themselves [7] and that there may be additional effects of disease interactions. A recent review identifies 14 studies that try to reduce the heterogeneity of multimorbidity [8], which can be done in two possible ways. First, one can start with the full complexity of multimorbidity and identify clusters of diseases that are often diagnosed together (“cluster method”) [9]. Second, one can start with single diseases and group them into disease combinations usually based on prevalence figures (“combination method”) [1].

Both methods have limitations. In using the combination method, the unit size (e.g. disease triads) does not necessarily match the number of conditions of a single person so that there are always more combinations than people in a data set. Regarding triads, for example, if a person has four diseases he/she will appear four times in the data set. The combination method does not show how combinations overlap in people, so that it is difficult to infer from combination-level to person-level. In contrast, using the cluster method, one cannot determine how diseases are associated within one cluster. For example, some diseases might only be in the same cluster because they are linked through a third disease, e.g. hypertension and renal insufficiency might be linked through diabetes. As clusters overlap, one might also ask which diseases serve as a bridge between clusters.

Therefore, this study aims at transcending the cluster and combination methods in order to depict which diseases are associated with each other on person-level within the clusters and which ones are responsible for the overlapping of multimorbidity clusters.

## Methods

### Data set

The analyses are based on ambulatory data of the *Gmünder ErsatzKasse*, a German statutory health insurance company with 1.7 million insurants (in 2008), which corresponds to 2.4% of the statutory insured population [10]. In Germany, about 90% of the total population is insured by statutory health insurance companies, because there is an obligation to be part of the statutory insurance system. The only exceptions are public servants (“Beamte”), clerics, professional soldiers, self-employed people (except artists and farmers), and people with an income over 52.000€/year who decide that they want to leave the statutory health insurance system. People in Germany who are not part of the statutory insurance system can be insured by private health insurance companies.

The *Gmünder ErsatzKasse* has a greater proportion of elderly male insurants than the general population of Germany, therefore, data analyses have to be adjusted for gender. The dataset contains pseudonymised data from every insured member of this company. The sample used for our analyses consists of all people aged 65 years and older who were continuously insured throughout the year 2006. The claims data set includes all diseases reported by ambulatory physicians. All problems managed by physicians within the statutory health insurance system have to be coded in ICD-10 (International Statistical Classification of Diseases and Related Health Problems, 10th Revision) and forwarded to the health insurance companies as regulated by §295(1) of the German Social Security Code *SGB V* and §44(3) of the Federal Collective Agreement within the statutory health insurance system in Germany [11]. As there is no limit to the number of diagnoses per patient that can be forwarded to health insurance companies, all diagnoses by ambulatory physicians are included in the data set.

The analysis of morbidity was based on a list of 46 defined diagnosis groups of chronic diseases (see below) derived from ICD-10 codes. The diagnoses were only counted if they were coded in at least three out of four quarters (three month periods) in 2006. This criterion was chosen in order to increase the validity of the diagnoses based on claims data by avoiding transitory or even accidental diagnoses. Prevalence, gender-specific rank order, and ICD-10 codes of the diagnosis groups have been published in another study [9].

The methods for compiling the list of 46 diagnosis groups have been described elsewhere in detail [1]. In short, we used the most frequent conditions documented in GP surgeries as listed in a panel survey of the Central Research Institute of Statutory Ambulatory Health Care in Germany (“ADT-Panel”) [12]. Chronicity of diagnoses was assessed using the report of the expert group on the implementation of a morbidity oriented risk adjustment scheme in the German Statutory Health Insurance [13]. In order to capture a comprehensive picture of the disease patterns in individual patients, we amended this list by all chronic conditions with a prevalence ≥ 1% in the age group ≥ 65 years in the data set of the *Gmünder ErsatzKasse* in 2006. ICD-10 codes were grouped together if diseases and syndromes had comparable pathophysiological mechanisms and if ICD codes of related disorders were supposed to be used ambiguously by coding physicians in clinical reality, respectively.

### Statistical analyses

The main feature of the statistical methods used in this study is a network analysis [14] which shows all associations between the selected diseases in graph format. The resulting “disease network” shows which diseases are often diagnosed together and how many associations to other diseases each disease has. The network analysis incorporates information from two previously published analyses: 1) triads identified by prevalence and observed/expected ratio [1] and 2) multimorbidity clusters from factor analysis [9].

The analyses were conducted in the multimorbid population, i.e. we included all patients who had at least three chronic conditions from the list of 46 diagnosis groups. We performed our analyses for both genders separately, because there are big differences between males and females in the prevalence rates of chronic conditions and we, therefore, wanted to allow for a possibly different association structure. To determine which diseases were associated with each other, we started with triads, i.e. combinations of three diseases. We used the prevalence as a criterion for relevance. Triads with a prevalence < 1% in the data set were excluded. The association between diseases was determined by observed/expected ratios (“O/E-ratios”). The expected prevalence of triads was calculated by multiplying the total prevalence of the single diseases within this triad by each other. The O/E-ratio was then estimated by dividing the observed by the expected prevalence of the triad. We defined diseases within a triad as associated with each other when the O/E-ratio was ≥ 2. For example, if we looked at the combination of hypertension (prevalence in the multimorbid male population: 69.4%), lipid metabolism disorders (48.0%) and chronic low back pain (41.0%), we expected a prevalence of 13.7% (0.694*0.480*0.410 = 0.137). We observed a prevalence of 13.9% for this combination in the data set. Therefore, the O/E-ratio in this case was 1.01 (0.139/0.137 = 1.01), which was below 2 and, therefore, the three diseases were not associated according to our definition and the triad was excluded from further analyses. The analyses for identifying relevant and associated triads were conducted using R version 3.0.1 [15].

For triads considered as relevant and associated, we extracted an edgelist, i.e. a list of all disease pairs contained within the selected triads. The edgelist was used to visualise the disease networks of both genders. The position of the diseases within the two-dimensional space was computed by a multidimensional scaling procedure. These analyses were conducted with the Stata 12.1 network analysis module Netplot by Rense Corton [14].

The results from this procedure were grouped (see next paragraph) using the multimorbidity clusters identified by factor analysis presented in a previous study [9]. In short, correlations between diagnosis groups were analysed by exploratory factor analysis based on a tetrachoric correlation matrix. We used an oblique (oblimin) rotation of factor loading matrices. The factors that resulted from this analysis could be interpreted as clusters of diagnosis groups frequently associated with each other. Factors were regarded as substantial if they had an eigenvalue of 1.0 or more. Diagnoses were assigned to a cluster if they had a factor loading of 0.25 or more on the pattern in charge.

As a next step of the analysis presented here, the single diseases were grouped into the multimorbidity clusters of “cardiovascular and metabolic disorders” and “anxiety, depression, somatoform disorders and pain”. The pattern “neuropsychiatric disorders” was not used as a grouping variable because we did not find a triad that represented three diseases from this pattern in either gender. However, the single neuropsychiatric diseases were still part of the data analysis. The relation between diseases and the multimorbidity cluster they were assigned to was pictured in two figures based on the Netplot graphs mentioned above.

We also calculated quantitative measures for the disease networks, which included node degree centrality (i.e. the number of observed edges of one disease in relation to the number of possible edges, which was used as a measure for the connectedness of a disease) and node betweenness centrality (i.e. the number of times that diseases served as a bridge in the shortest pathway between other diseases, which was used as a measure for the potential influence of a disease on the distribution of other diseases in one person). Additionally, we also calculated the average node betweenness centrality as a (negative) measure for the total extent of accumulation of diseases within the networks. These analyses were performed by Visone 2.7.3.

In order to provide greater confidence in the study results, we replicated all analyses with the ambulatory data of the *Gmünder ErsatzKasse* from 2004. As the prevalence of most chronic conditions was significantly lower in 2004, we chose to lower the prevalence criterion for triads from 1.0% to 0.85% in this data set, which resulted in a comparable number of eligible triads as in 2006. All other methods, i.e. the methods for data preparation, factor analyses, extraction of the edgelists, and network analysis, as well as the O/E-ratio used for triad inclusion, were the same as in 2006.

### Ethics statement

The research presented in this paper was conducted according to the principles expressed in the Declaration of Helsinki. We did not have to obtain informed consent because our research was based on insurance claims data and the data set was analysed anonymously (as regulated by German law pursuant to §75 *SGB X*). The study was approved by the Ethics Committee of the Medical Association of Hamburg (approval no. PV3057). The approval included the waiver of consent.

## Results

We found a total of 149,280 patients in our 2006 data set of which 98,619 (66.1%) were defined as multimorbid because they had 3 or more chronic conditions from our list of 46 diagnosis groups. The distribution of age, gender, and the number of chronic conditions in the multimorbid population is shown in Table 1. 43,632 (44.2%) of these patients were female and 54,987 (55.8%) were male. The triads by multimorbidity clusters that had previously been identified by factor analysis are shown in Tables 2 and 3. We found 13,460 triads of chronic conditions in the female population of which 932 had a prevalence ≥ 1% and 57 of these also had an O/E ratio ≥ 2 (cf. Table 2). In the male population, we found 14,105 triads of which 830 had a prevalence ≥ 1% and 51 of these also had an O/E ratio ≥ 2 (cf. Table 3). In total, the selected triads of females consisted of 31 chronic conditions with 200 edges and the selected triads of males included 29 chronic conditions with 174 edges.

**Table 1:** Age and the number of diagnosis groups by gender in the multimorbid* population of 2006 and 2004

	2006		2004	
	Females	Males	Females	Males
Number of patients	43,632 (44.2%)	54,987 (55.8%)	32,369 (44.6%)	40,179 (55.4%)
Age: mean ± sd	73.3 ± 6.8	72.2 ± 5.9	73.6 ± 6.8	72.3 ± 5.9
Chronic conditions: mean ± sd	6.0 ± 2.7	5.9 ± 2.6	5.7 ± 2.6	5.6 ± 2.5

sd = standard deviation; *patients are defined as multimorbid if they have ≥ 3 chronic conditions.

**Table 2:** **Triads with a prevalence ≥ 1% and an observed/expected ratio ≥ 2 by multimorbidity clusters in the female population with ≥ 3 chronic conditions (n = 43.632)**

Cardiovascular and metabolic disorders	O/E-ratio	Prevalence in %
Diabetes mellitus + Hyperuricemia/Gout + Liver diseases	3.8	1.0
Diabetes mellitus + Obesity + Hyperuricemia/Gout	3.7	1.4
Hypertension + Hyperuricemia/Gout + Renal insufficiency	3.7	1.2
Chronic ischemic heart disease + Cardiac arrhythmias + Cardiac insufficiency	3.7	1.1
Lipid metabolism disorders + Hyperuricemia/Gout + Liver diseases	3.1	1.6
Diabetes mellitus + Chronic ischemic heart disease + Cardiac insufficiency	3.0	1.4
Diabetes mellitus + Chronic ischemic heart disease + Neuropathies	2.9	1.0
Lipid metabolism disorders + Cardiac arrhythmias + Cardiac valve disorders	2.8	1.0
Hypertension + Cardiac arrhythmias + Cardiac valve disorders	2.7	1.5
Diabetes mellitus + Chronic ischemic heart disease + Hyperuricemia/Gout	2.7	1.4
Severe vision reduction + Diabetes mellitus + Neuropathies	2.7	1.2
Diabetes mellitus + Chronic ischemic heart disease + Atherosclerosis/PAOD	2.7	1.2
Lipid metabolism disorders + Obesity + Hyperuricemia/Gout	2.6	1.8
Lipid metabolism disorders + Diabetes mellitus + Hyperuricemia/Gout	2.3	3.0
Hypertension + Obesity + Hyperuricemia/Gout	2.3	2.3
Hypertension + Hyperuricemia/Gout + Liver diseases	2.3	1.8
Lipid metabolism disorders + Chronic ischemic heart disease + Cardiac valve disorders	2.3	1.0
Hypertension + Chronic ischemic heart disease + Renal insufficiency	2.2	1.2
Hypertension + Atherosclerosis/PAOD + Cerebral ischemia/Chronic stroke	2.2	1.2
Lipid metabolism disorders + Hyperuricemia/Gout + Cardiac insufficiency	2.2	1.2
Hypertension + Chronic ischemic heart disease + Cardiac insufficiency	2.1	2.6
Lipid metabolism disorders + Chronic ischemic heart disease + Atherosclerosis/PAOD	2.1	1.8
Diabetes mellitus + Chronic ischemic heart disease + Cardiac arrhythmias	2.1	1.5
Hypertension + Diabetes mellitus + Renal insufficiency	2.1	1.5
Diabetes mellitus + Chronic ischemic heart disease + Obesity	2.1	1.3
Lipid metabolism disorders + Obesity + Liver diseases	2.1	1.3
Lipid metabolism disorders + Hyperuricemia/Gout + Atherosclerosis/PAOD	2.1	1.2
Lipid metabolism disorders + Liver diseases + Atherosclerosis/PAOD	2.1	1.0
Hypertension + Diabetes mellitus + Obesity	2.0	4.6
Lipid metabolism disorders + Chronic ischemic heart disease + Hyperuricemia/Gout	2.0	1.9
Hypertension + Chronic ischemic heart disease + Cardiac valve disorders	2.0	1.3
Severe vision reduction + Diabetes mellitus + Atherosclerosis/PAOD	2.0	1.1
**Anxiety, depression, somatoform disorders and pain**	**O/E**	**%**
Chronic low back pain + Depression + Somatoform disorders	2.6	1.7
Chronic low back pain + Asthma/COPD + Allergies	2.4	1.4
Chronic low back pain + Intestinal diverticulosis + Chronic gastritis/GERD	2.4	1.1
Chronic low back pain + Depression + Insomnia	2.3	1.5
Joint arthrosis + Depression + Somatoform disorders	2.3	1.1
Chronic low back pain + Depression + Dizziness	2.3	1.0
Joint arthrosis + Depression + Insomnia	2.3	1.0
Chronic low back pain + Somatoform disorders + Chronic gastritis/GERD	2.2	1.2
Chronic low back pain + Lower limb varicosis + Hemorrhoids	2.1	1.2
Chronic low back pain + Gynaecological problems + Urinary incontinence	2.1	1.2
Chronic low back pain + Chronic gastritis/GERD + Insomnia	2.1	1.1
Chronic low back pain + Gynaecological problems + Somatoform disorders	2.0	1.2
Depression + Osteoporosis + Chronic gastritis/GERD	2.0	1.1
**Both multimorbidity patterns**	**O/E**	**%**
Joint arthrosis + Obesity + Hyperuricemia/Gout	2.5	1.2
Chronic ischemic heart disease + Lower limb varicosis + Cardiac insufficiency	2.5	1.0
Joint arthrosis + Chronic ischemic heart disease + Cardiac insufficiency	2.4	1.5
Chronic low back pain + Hyperuricemia/Gout + Liver diseases	2.3	1.2
Hypertension + Depression + Anxiety	2.2	1.0
Chronic low back pain + Obesity + Hyperuricemia/Gout	2.1	1.5
Diabetes mellitus + Obesity + Lower limb varicosis	2.1	1.5
Joint arthrosis + Diabetes mellitus + Neuropathies	2.1	1.4
Chronic low back pain + Depression + Neuropathies	2.1	1.4
Chronic low back pain + Neuropathies + Chronic gastritis/GERD	2.1	1.2
Joint arthrosis + Obesity + Lower limb varicosis	2.0	2.0
Lipid metabolism disorders + Asthma/COPD + Allergies	2.0	1.1

O/E: observed/expected ratio; PAOD: peripheral arterial occlusive disease; COPD: chronic obstructive pulmonary disease; GERD: gastroesophageal reflux disease.

**Table 3:** **Triads with a prevalence ≥ 1% and an observed/expected ratio ≥ 2 by multimorbidity clusters in the male population with ≥ 3 chronic conditions (n = 54.987)**

Cardiovascular and metabolic disorders	O/E-ratio	Prevalence in %
Obesity + Hyperuricemia/Gout + Liver diseases	3.5	1.3
Chronic ischemic heart disease + Cardiac arrhythmias + Cardiac insufficiency	3.3	1.7
Diabetes mellitus + Atherosclerosis/PAOD + Neuropathies	3.3	1.4
Chronic ischemic heart disease + Cardiac arrhythmias + Cardiac valve disorders	3.2	1.2
Diabetes mellitus + Obesity + Liver diseases	3.0	1.5
Severe vision reduction + Diabetes mellitus + Neuropathies	3.0	1.5
Chronic ischemic heart disease + Atherosclerosis/PAOD + Renal insufficiency	2.9	1.2
Chronic ischemic heart disease + Atherosclerosis/PAOD + Cardiac insufficiency	2.9	1.2
Diabetes mellitus + Atherosclerosis/PAOD + Renal insufficiency	2.6	1.1
Chronic ischemic heart disease + Hyperuricemia/Gout + Renal insufficiency	2.5	1.5
Chronic ischemic heart disease + Cardiac arrhythmias + Renal insufficiency	2.4	1.3
Chronic ischemic heart disease + Atherosclerosis/PAOD + Cerebral ischemia/Chronic stroke	2.4	1.3
Diabetes mellitus + Atherosclerosis/PAOD + Cerebral ischemia/Chronic stroke	2.4	1.2
Hypertension + Renal insufficiency + Cardiac insufficiency	2.4	1.1
Lipid metabolism disorders + Hyperuricemia/Gout + Liver diseases	2.3	3.5
Diabetes mellitus + Hyperuricemia/Gout + Liver diseases	2.3	2.3
Diabetes mellitus + Obesity + Hyperuricemia/Gout	2.3	1.9
Lipid metabolism disorders + Obesity + Liver diseases	2.3	1.7
Diabetes mellitus + Hyperuricemia/Gout + Renal insufficiency	2.3	1.4
Diabetes mellitus + Cardiac arrhythmias + Cardiac insufficiency	2.3	1.2
Hypertension + Urinary incontinence + Cancers	2.3	1.1
Lipid metabolism disorders + Hyperuricemia/Gout + Renal insufficiency	2.2	2.1
Diabetes mellitus + Chronic ischemic heart disease + Cardiac insufficiency	2.2	1.8
Hypertension + Cardiac arrhythmias + Cardiac valve disorders	2.1	1.8
Diabetes mellitus + Chronic ischemic heart disease + Renal insufficiency	2.1	1.7
Lipid metabolism disorders + Cardiac arrhythmias + Cardiac valve disorders	2.1	1.2
Chronic ischemic heart disease + Hyperuricemia/Gout + Cardiac insufficiency	2.1	1.2
Hyperuricemia/Gout + Liver diseases + Atherosclerosis/PAOD	2.1	1.1
Hypertension + Hyperuricemia/Gout + Renal insufficiency	2.0	2.7
Lipid metabolism disorders + Obesity + Hyperuricemia/Gout	2.0	2.7
Hypertension + Obesity + Liver diseases	2.0	2.3
Hypertension + Cardiac arrhythmias + Cardiac insufficiency	2.0	2.3
Diabetes mellitus + Chronic ischemic heart disease + Neuropathies	2.0	1.7
Lipid metabolism disorders + Atherosclerosis/PAOD + Cerebral ischemia/Chronic stroke	2.0	1.6
Chronic low back pain + Cardiac arrhythmias + Cardiac valve disorders	2.0	1.0
**Anxiety, depression, somatoform disorders and pain**	**O/E**	**%**
Chronic low back pain + Hemorrhoids + Chronic gastritis/GERD	2.5	1.0
Chronic low back pain + Joint arthrosis + Hemorrhoids	2.3	1.6
Chronic low back pain + Prostatic hyperplasia + Sexual dysfunction	2.3	1.2
Chronic low back pain + Joint arthrosis + Lower limb varicosis	2.2	2.5
Chronic low back pain + Joint arthrosis + Intestinal diverticulosis	2.2	1.4
Chronic low back pain + Depression + Chronic gastritis/GERD	2.2	1.1
Chronic low back pain + Prostatic hyperplasia + Hemorrhoids	2.1	1.6
Chronic low back pain + Joint arthrosis + Insomnia	2.1	1.3
Chronic low back pain + Joint arthrosis + Severe hearing loss	2.1	1.1
**Both multimorbidity patterns**	**O/E**	**%**
Chronic ischemic heart disease + Asthma/COPD + Cardiac insufficiency	2.5	1.0
Chronic low back pain + Joint arthrosis + Neuropathies	2.2	1.8
Chronic low back pain + Obesity + Liver diseases	2.2	1.4
Chronic low back pain + Atherosclerosis/PAOD + Neuropathies	2.1	1.1
Joint arthrosis + Obesity + Hyperuricemia/Gout	2.0	1.4
Hyperuricemia/Gout + Liver diseases + Asthma/COPD	2.0	1.1
Hyperuricemia/Gout + Prostatic hyperplasia + Renal insufficiency	2.0	1.1

O/E: observed/expected ratio; PAOD: peripheral arterial occlusive disease; COPD: chronic obstructive pulmonary disease; GERD: gastroesophageal reflux disease.

**Figure 1:**
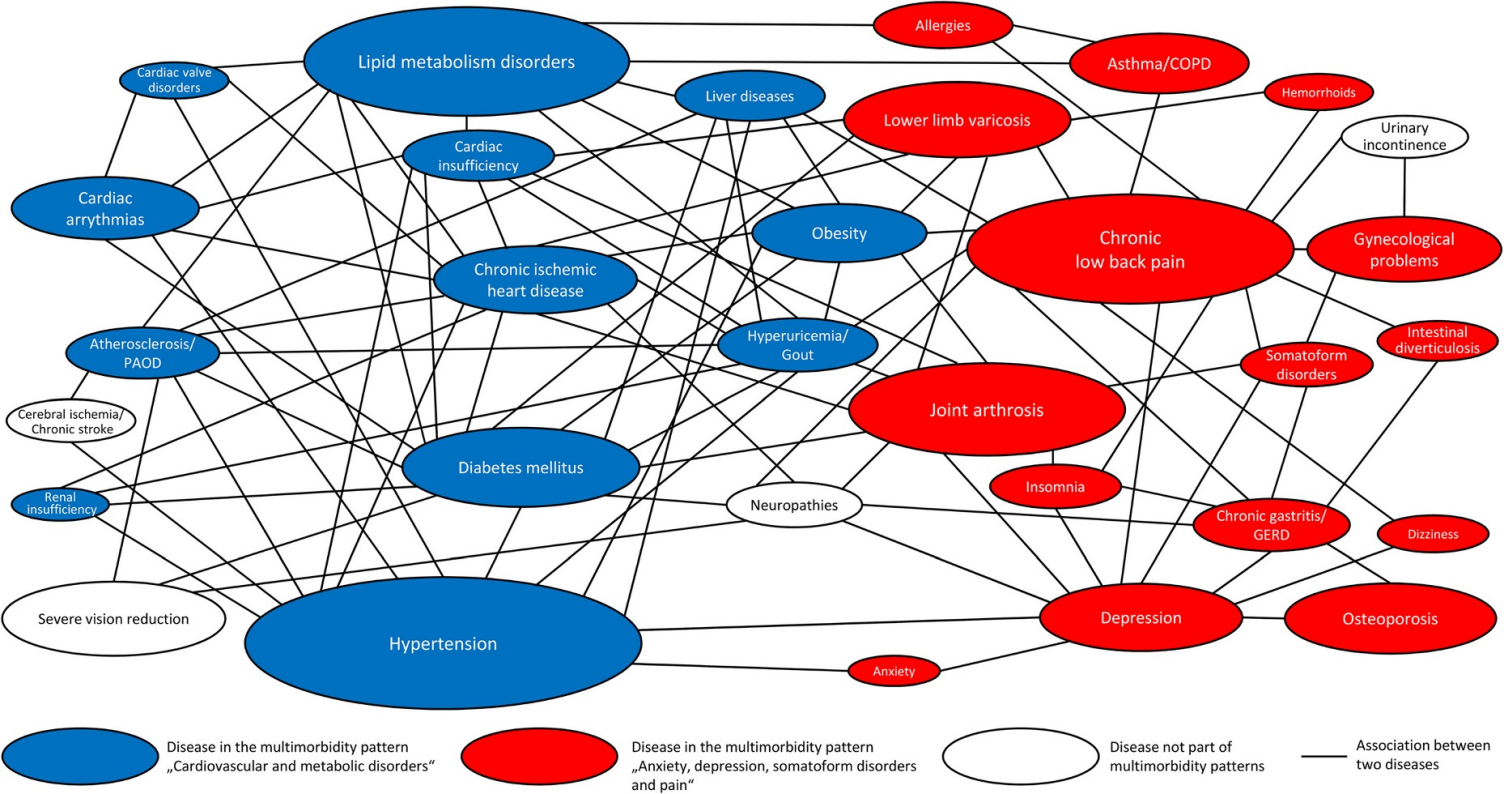
**Disease associations in multimorbidity clusters based on triads with a prevalence ≥ 1% and an observed/expected ratio ≥ 2 in the female population with ≥ 3 chronic conditions.**

**Figure 2:**
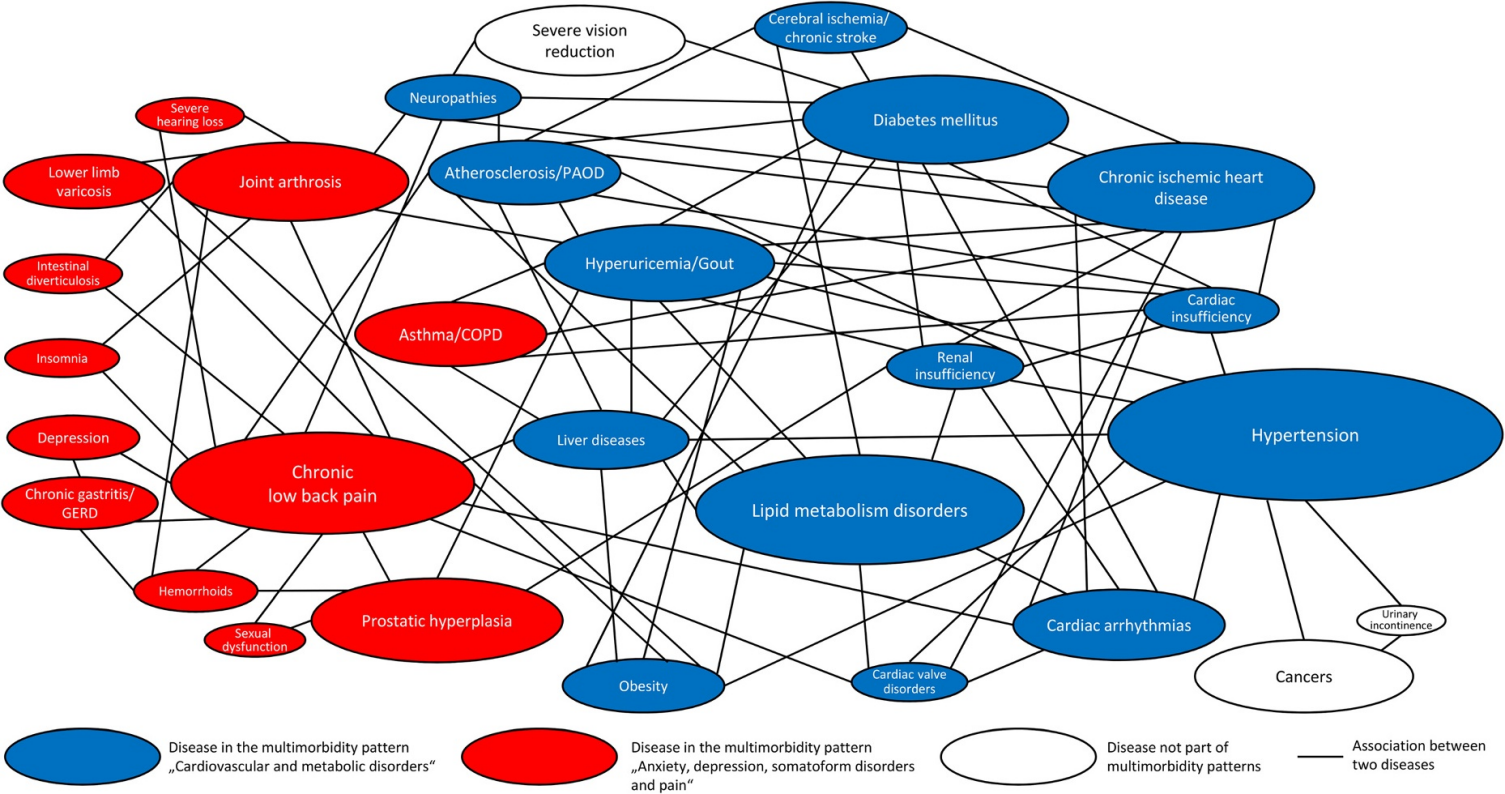
**Disease associations in multimorbidity clusters based on triads with a prevalence ≥ 1% and an observed/expected ratio ≥ 2 in the male population with ≥ 3 chronic conditions.**

The prevalence, the number of edges as well as degree and betweenness centrality of chronic conditions are shown in Table 4. In both genders, the condition with the highest number of edges was chronic low back pain (16 edges in females and males), i.e. chronic low back pain was associated with 16 other chronic conditions, which corresponded to 8.0% of all possible connections (degree centrality) in females and 9.2% in males. Chronic low back pain also was the most central disease in the networks of both genders in terms of being the most important mediator of connections between other chronic conditions (betweenness centrality). Another disease with high betweenness centrality in both genders was hypertension. Depression also was an important mediator of disease connections in females, but not in males. The average level of accumulation in the female disease network (average node betweenness) was 3.2%, while the male disease network had a slightly higher average node betweenness of 3.4% and thus was slightly less accumulated than the female disease network.Figure 1 shows the disease associations in the multimorbid female population and Figure 2 shows them in the multimorbid male population. In these figures, each ellipse represents a chronic condition and the surface area of each ellipse is proportional to the prevalence of the disease. Each line represents an association between two chronic conditions. For example, the triad “hypertension + urinary incontinence + cancers” in the male population is represented by three ellipses and the three lines between these ellipses in the lower right corner of Figure 2. These figures can be interpreted that two diseases linked by an edge are often diagnosed together on person-level. For this interpretation, only direct links are relevant, e.g. in the female population, chronic low back pain is linked to somatoform disorders and dizziness, but there is no edge between somatoform disorders and dizziness. This means that chronic low back pain is often diagnosed together with a somatoform disorder, and chronic low back pain also is often diagnosed together with dizziness, but the diagnosis of dizziness does not often occur in conjunction with a somatoform disorder diagnosis.

**Table 4:** **Prevalence, number of edges, degree and betweenness centrality of diseases by gender and multimorbidity clusters in the population with ≥ 3 chronic conditions**

ADS and pain	Prevalence in patients			Number of edges (degree centrality)		Betweenness centrality	
	Total	Females	Males	Females	Males	Females	Males
Chronic low back pain	43.7%	47.1%	41.0%	16 (8.0%)	16 (9.2%)	28.4%	32.2%
Joint arthrosis	28.7%	33.6%	24.8%	10 (5.0%)	9 (5.2%)	4.9%	5.7%
Lower limb varicosis	16.4%	22.7%	11.5%	7 (3.5%)	2 (1.1%)	3.1%	0.0%
Prostatic hyperplasia	*	-	28.1%	-	5 (2.9%)	-	2.6%
Asthma/COPD	15.3%	14.1%	16.3%	3 (1.5%)	4 (2.3%)	0.4%	0.1%
Depression	12.4%	18.1%	7.8%	10 (5.0%)	2 (1.1%)	11.9%	0.0%
Chronic gastritis/GERD	10.8%	10.8%	10.8%	7 (3.5%)	3 (1.7%)	2.7%	0.1%
Osteoporosis	10.7%	19.7%	3.6%	2 (1.0%)	-	0.0%	-
Gynaecological problems	*	16.6%	-	3 (1.5%)	-	0.1%	-
Allergies	7.3%	8.5%	6.3%	3 (1.5%)	-	0.4%	-
Insomnia	6.6%	7.6%	5.8%	4 (2.0%)	2 (1.1%)	0.2%	0.0%
Intestinal diverticulosis	6.3%	6.4%	6.2%	2 (1.0%)	2 (1.1%)	0.0%	0.0%
Hemorrhoids	6.1%	5.2%	6.8%	2 (1.0%)	4 (2.3%)	0.0%	0.4%
Somatoform disorders	5.9%	7.7%	4.5%	5 (2.5%)	-	1.1%	-
Severe hearing loss	4.6%	3.8%	5.2%	-	2 (1.1%)	-	0.0%
Dizziness	4.2%	5.4%	3.4%	2 (1.0%)	-	0.0%	-
Anxiety	2.5%	3.7%	1.6%	2 (1.0%)	-	0.0%	-
Sexual dysfunction	*	-	4.5%	-	2 (1.1%)	-	0.0%
**Cardiovascular and metabolic**							
Hypertension	69.3%	69.3%	69.4%	13 (6.5%)	9 (5.2%)	12.0%	12.0%
Lipid metabolism disorders	47.4%	46.7%	48.0%	11 (5.5%)	8 (4.6%)	5.8%	1.4%
Diabetes mellitus	28.5%	24.9%	31.4%	14 (7.0%)	11 (6.3%)	5.8%	5.0%
Chronic ischemic heart disease	25.7%	18.2%	31.6%	13 (6.5%)	10 (5.7%)	5.1%	2.8%
Hyperuricemia/Gout	18.1%	11.3%	23.5%	11 (5.5%)	12 (6.9%)	5.2%	7.8%
Cardiac arrhythmias	18.0%	15.5%	20.0%	6 (3.0%)	8 (4.6%)	0.2%	4.3%
Atherosclerosis/PAOD	13.7%	10.3%	16.4%	8 (4.0%)	10 (5.7%)	2.6%	5.7%
Obesity	12.4%	13.4%	11.7%	9 (4.5%)	7 (4.0%)	2.2%	3.8%
Liver diseases	12.0%	9.9%	13.7%	7 (3.5%)	8 (4.6%)	2.0%	4.5%
Cardiac insufficiency	9.0%	10.1%	8.1%	8 (4.0%)	8 (4.6%)	1.0%	1.3%
Cerebral ischemia/Chronic stroke	9.0%	7.3%	10.3%	2 (1.0%)	4 (2.3%)	0.0%	0.1%
Neuropathies	8.2%	8.1%	8.2%	7 (3.5%)	6 (3.4%)	4.6%	3.9%
Renal insufficiency	6.5%	4.2%	8.3%	4 (2.0%)	9 (5.2%)	0.0%	2.6%
Cardiac valve disorders	5.7%	5.2%	6.0%	4 (2.0%)	5 (2.9%)	0.1%	2.2%
**No multimorbidity pattern**							
Severe vision reduction	20.6%	22.0%	19.6%	3 (1.5%)	2 (1.1%)	0.1%	0.0%
Cancers	18.4%	14.8%	21.2%	-	2 (1.1%)	-	0.0%
Urinary incontinence	5.1%	7.1%	3.5%	2 (1.0%)	2 (1.1%)	0.0%	0.0%

ADS: Anxiety, depression and somatoform disorders; PAOD: peripheral arterial occlusive disease; COPD: chronic obstructive pulmonary disease; GERD: gastro-esophageal reflux disease; *gender-specific disease.

Our replication analysis in the 2004 data set showed similar results as in 2006. A total of 123,224 patients were included in these analyses of which 72,548 were multimorbid according to our definition. Age, gender, and the number of chronic conditions of this population are shown in Table 1. Regarding the factor analysis, we found a very similar association structure in 2004 with the same three clusters extracted for males and females as in 2006. The only notable difference in the analyses presented here was that the ADS and pain cluster in the male population of 2004 did not include prostatic hyperplasia and sexual dysfunction. Instead, one additional multimorbidity cluster with prostatic cancer and related disorders had been extracted. Except for that, only minor differences between both years were found in the factor analyses.

In 2004 we found 957 triads with a prevalence ≥ 0.85% in the female population and 56 of these also had an O/E ratio ≥ 2. In the male population, we found 819 triads with a prevalence ≥ 0.85% and 49 of these also had an O/E ratio ≥ 2. In total, the selected triads of females consisted of 29 chronic conditions with 194 edges and the selected triads of males included 27 chronic conditions with 164 edges. The edgelists of 2004 produced comparable figures of disease networks as in 2006 with a clear separation between the multimorbidity clusters: 1) ADS and pain and 2) cardiovascular and metabolic disorders. Degree and betweenness centrality in the cluster “ADS and pain” were comparable to 2006, as chronic low back pain also had the highest degree and betweenness centrality of all nodes and depression still had the second highest betweenness centrality in females in this cluster. The only notable differences related to betweenness centrality in the cluster “cardiovascular and metabolic disorders”, as hypertension did not constitute an important mediator between chronic conditions in any gender, but was replaced in 2004 by hyperuricemia/gout in males and chronic ischemic heart disease in females.

## Discussion

### Associations between diseases

This is the first study that uses a systematic approach to integrate the two current methods to determine multimorbidity clusters in the older population and, therefore, includes information about the association between single diseases on person-level. It shows epidemiologically relevant associations between diseases in graph format and also gives information about the number of connections of one disease to other diseases as well as how much potential influence it has on the distribution of other diseases in one person. This information can provide a basis for selecting relevant comorbidities when designing clinical practice guidelines or deciding which comorbidity-based treatment recommendations are relevant.

The chronic condition with the highest number of associations was chronic low back pain, which also appeared to be by far the most important mediator of connections between other chronic conditions. High numbers of edges were also found in both genders for joint arthrosis and for several metabolic (hyperuricemia/gout, diabetes mellitus) and cardiovascular diseases (hypertension, chronic ischemic heart disease). The results presented here suggest that out of these conditions, hypertension is the one that serves as the most important bridge between other diseases. However, as our replication analysis shows, this result changes over time. In the 2004 data set, hyperuricemia/gout (males) and chronic ischemic heart diseases (females) seemed to serve as important bridges instead. Among women, depression was also characterized by many edges and it also seemed to be mediating many other disease connections. It should be noted that diseases with a higher prevalence did not necessarily have a higher degree centrality or betweenness centrality than diseases with a lower prevalence (e.g. in the male population, hyperuricemia/gout had only half the prevalence of lipid metabolism disorders but 50% more edges and a much higher betweenness centrality).

An association of low back pain with a large number of chronic conditions had been reported before, e.g. with cardiovascular diseases [16] as well as psychiatric diagnoses and pain syndromes [17]. In a previous study, we found that the prevalence of chronic low back pain increased more than expected by chance with the number of comorbidities one person had [18]. For this reason, chronic low back pain might be more often the result of and not be the cause for other diseases. Low back pain could also be part of some general frailty pattern [16]. Raspe et al. suggest that back pain undergoes a unidirectional process of chronification towards a complex pain syndrome including other types of pain as well as functional and emotional impairments [19]. Another explanation might be that chronic low back pain is often found as a secondary diagnosis when multimorbid patients consult a physician for other reasons. E.g. Waxman et al. identified that 71% of patients with back pain did not see their physician for this reason and that depression symptoms had a higher impact on consultation rates for back pain than pain characteristics [20].

As stated above, we also detected a multitude of associations between disorders that belonged to the metabolic syndrome (i.e. obesity, hypertension, lipid metabolism disorders and diabetes mellitus [21]), hyperuricemia/gout and cardiovascular diseases (i.e. hypertension, atherosclerosis, chronic ischemic heart disease) in both genders. Such co-occurrences have been well documented. For example, the relative excess risk for cardiovascular disease is estimated to be two to eight times higher in people with diabetes than in non-diabetic individuals, when adjusted for age, sex, and ethnicity [22]. A main reason for this association might be an overlapping of behavioural and/or genetic risk factors for diabetes and cardiovascular disease [21]. It has also been implied that diabetes mellitus and gout share the same risk factors [23].

### Multimorbidity clusters

Except for asthma/COPD, we found the same structure of multimorbidity clusters in the network analysis of triadic disease combinations in both genders as in the previously published factor analysis [9]. Reasons for these associations are discussed in the literature. For example, the association between respiratory and cardiovascular disease might be due to systemic inflammation, chronic infections, shared risk factors (such as smoking) or other undefined factors [24].

The network analysis also showed some associations within the multimorbidity clusters that were overlooked by factor analysis. Regarding network analysis, this relates particularly to neuropathies and severe vision reduction associated with the multimorbidity cluster of cardiovascular and metabolic disorders in both genders As both disorders are frequent complications of diabetes mellitus [25], this seems to be a plausible extension of this pattern. In females, the pattern of cardiovascular and metabolic disorders was amended by cerebral ischemia/chronic stroke, which had already been included in this pattern for the male population in the factor analysis.

We found three reasons for overlapping phenomena between the multimorbidity clusters cardiovascular and metabolic disorders and ADS and pain. First, there might be medical explanations, such as shared risk factors, causation and complications, e.g. the association between diseases that constitute the metabolic syndrome and musculoskeletal disorders. There is evidence that obesity and associated diseases, like diabetes mellitus, might be risk factors for low back pain [26, 27] and osteoarthritis [28, 29]. There is also some evidence that anxiety and depression might lead to hypertension [30] and associated disorders. A second group of reasons for overlapping multimorbidity clusters might be related to utilization patterns of the different medical disciplines in ambulatory care. For example, an urologist consulted for prostatic hyperplasia might regularly test for renal insufficiency or hyperuricemia and, therefore, the prevalence of these diagnoses might be higher in patients with prostatic hyperplasia. Third, there is no obvious explanation for some associations between diseases of different patterns, e.g. the triad of cardiac valve disorders, cardiac arrhythmias and chronic low back pain. Research is needed to identify the mechanisms for this type of overlapping if these relations are confirmed.

### Gender differences

Diseases seem to be more associated to each other in the female population (with 15% more edges and a slightly lower average node betweenness) than in the male population. This phenomenon can be partly explained by an 11% higher number of triads with a prevalence ≥ 1% in females than in males. However, we generally found a comparable disease network in both genders. In the cluster of cardiovascular and metabolic disorders, we found a high concordance between both genders. Females and males both had diabetes mellitus, chronic ischemic heart disease and hyperuricemia/gout among the five most associated diseases and comparable disease associations in this cluster.

Gender differences were more prominent in the multimorbidity cluster of ADS and pain. Although chronic low back pain and joint arthrosis were the two most associated diseases in both genders, women generally had more associations with psychiatric/psychosomatic disorders, e.g. the diagnoses anxiety and somatoform disorders were only present in the disease matrix of the female population. This also applied for cases of depression which had a comparably high degree and betweenness centrality in females, but not in males. The main reason for these differences might be due to gender-dependent prevalence rates, e.g. the prevalence of depression was more than twice as high in the female than in the male population in our study. These gender-differences related to depression are confirmed by a wide range of other studies [31]. Although sometimes influenced by artefacts, like reporting bias, gender-differences in depression can be considered to be a genuine phenomenon [21].

### Strengths and weaknesses

This study used a network analysis to combine results from factor analysis and observed/expected ratios of disease combinations in order to create a pattern-specific disease network. The methods were well matched as multimorbidity clusters from factor analysis could also be found in the network analysis and some diseases were identified that had been overlooked by factor analysis. The figures provided a systematic overview of all disease associations that were prevalent and more frequent than expected by chance. The analyses were based on triads of diseases because multimorbidity was defined by a three disease criterion. For this reason, associations that occurred only between two diseases were not considered.

A strength of our approach lies in the comprehensive selection of diseases by including all highly prevalent chronic conditions (≥1% in the age group 65+) into our diagnosis groups and applying them to our disease matrix. Unfortunately, a more recent data set than 2006 was not available for these analyses. However, we assume that the associations between diseases that we found are, for the most part, stable over time, thus results based on newer data will probably be similar. To provide more confidence in our results, we did a replication analysis with the 2004 data set of the same insurance company, which used the same methods as our 2006 analyses, except for a lowered prevalence criterion for triad inclusion. The reason for this decision was a much higher prevalence of the most chronic conditions in 2006 compared to 2004, probably due to changes in the documentation behaviour of ambulatory physicians. This phenomenon had been reported before, e.g. Uijen and Lisdonk found a 60% increase in the number of diagnoses of chronic conditions in the Netherlands between 1985 and 2005 [32]. This trend could probably also be observed if a more recent data set was used, which might mean that the prevalence criterion for triad inclusion needed to be increased in later years in order to keep results comparable. Our replication analysis confirmed the results presented here. However, there are generally great differences concerning the identification of multimorbidity clusters in different studies [8], so that there is still need for replication in other countries and populations.

It should also be noted that the study is based on diagnoses and not on diseases. Although accidental and transitory diagnoses were excluded, in some cases, diagnoses might be imprecise, ambiguous, or incomplete because they were not clinically verified by trained professionals. This is a general problem with insurance claims data [33], but in our view, the benefits of claims data outweigh their disadvantages: We are provided with a large unselected population group, representing real-world conditions, including people living in protected institutions/nursing homes, as well as frail individuals, and the oldest-old, i.e. groups of patients frequently not included in survey and field studies. In choosing insurance claims data, we also avoided selection bias concerning service providers, and there is no recall bias concerning diagnosis data. However, as results from multimorbidity studies might differ depending on the data set used [34], a replication of our study using other data sources should also be useful.

## Conclusion

Using the current methods for analysing associations between diseases, we knew the clustering of diseases and frequent disease combinations, but we missed the complete picture of multimorbidity, because the internal structure of the clusters and the linking between disease combinations and clusters were still unknown. The results presented here constitute the first attempt to respond to this academic vault. We are able to show which diseases are associated with each other in our data set, to which multimorbidity clusters the diseases are assigned and which diseases are responsible for overlapping multimorbidity clusters.

However, we have to keep in mind that this approach focuses on statistically selected diseases and that most patients suffer from more diseases than those represented in our analyses. For this reason, multimorbidity cannot be grasped completely. Nevertheless, our methodological approach might prove a good starting point for further analyses and guideline developers might find our approach useful as a basis for discussing which comorbidity should be addressed in clinical practice guidelines.
